# Impact of wearable technology on psychosocial factors of osteoarthritis management: a qualitative study

**DOI:** 10.1136/bmjopen-2015-010064

**Published:** 2016-02-03

**Authors:** Athina Belsi, Enrica Papi, Alison H McGregor

**Affiliations:** 1 Department of Surgery and Cancer, Imperial College London, St Mary's Campus, London, UK; 2 Department of Surgery and Cancer, Imperial College London, Charing Cross Hospital, London, UK

**Keywords:** Rehabilitation, wearable technology, Osteoarthritis, Focus groups

## Abstract

**Objectives:**

To identify the impact the use of wearable technology could have in patients with osteoarthritis in terms of communication with healthcare providers and patients’ empowerment to manage their condition.

**Design:**

Qualitative study using focus groups with patients with osteoarthritis; data from patients’ responses were analysed using Framework Methodology.

**Participants:**

21 patients with knee osteoarthritis from the London area (age range 45–65 years) participated in a total of four focus groups. Recruitment continued until data saturation.

**Setting:**

The study was conducted in a university setting.

**Results:**

Patients’ responses suggested a positive attitude on the impact wearable technology could have on the management of osteoarthritis. It was perceived that the use of wearable devices would benefit patients in terms of feeling in control of their condition, providing them with awareness of their progress, empowering in terms of self-management and improving communication with their clinician.

**Conclusions:**

This paper suggests positive patient perspectives on the perceived benefits wearable technology could have on the management of osteoarthritis. The data that could be collected with the use of wearable technology could be beneficial both to patients and clinicians. The information obtained from this study suggests that introducing wearable technology into patient-centred care could enhance patient experience in the field of osteoarthritis and beyond.

**Strengths and limitations of this study**

- This study explores an under-investigated area: patients’ views on the potential benefits wearable technology can have in empowering them to manage their osteoarthritis and improve communication with their clinicians.
- Qualitative methodology using focus groups offered the opportunity for a rich and deep insight into patients’ views.
- All the patients came from the London area and had not previously used the monitoring device.

## Introduction

The introduction and increasing use of wearable technology, intended as a small lightweight body-worn sensor, in health services has been linked to a shift from paternalistic to more collaborative approaches in the provision of healthcare:^1^ the latter become more patient-accessible and patient-centred, rather than provider-focused, while patients can be more actively involved in their own care and understand their health needs better.^2^ Wearable technology has also been associated with reduced healthcare costs, while at the same time it has the potential to improve the quality of health services provided, as it enables gathering accurate and personalised patient data that could inform treatment planning.^3^ Improvements in patients’ quality of life have also been identified as another outcome;^4^ wearable technology permits continuous monitoring, while the patients can carry on with their everyday activities, rather than being confined in a hospital setting.

In addition, it has been recognised that new technologies in healthcare have the potential to change the dynamics in patients’ monitoring and managing their own health,^5^ ^6^ while at the same time there is the potential for clinical and economic benefits.^7^

Especially in chronic conditions, including diabetes, chronic pain and back pain, it has been suggested that introducing medical technologies in the patients’ daily life can enhance empowerment and self-management.^2^ ^8^ Barlow *et al*^2^ have discussed that the latter have been found to be beneficial in terms of knowledge, behavioural change and self-efficacy and to have an overall positive effect on patients’ well-being. Indeed, in on-going conditions after the initial diagnosis and assessment, patients often have to take responsibility for self-management and monitoring their illness.^9^ As a result, providing patients with feedback on the progress of their health could potentially empower them to assume increased autonomy and control. This has actually been found beneficial in a range of conditions including teenagers with diabetes,^10^ hypertensive adults^11^ as well as increasing confidence in self-management abilities for patients with asthma.^9^ ^12^

Wearable technologies have also gained recent interest as new tools for tracking daily information related to activities performed and for measuring human movements.^13^ As such they offer the possibility to be used in the context of rehabilitation allowing daily functional limitations and joint weaknesses to be monitored while permitting patient engagement and motivation to maintain an active lifestyle or facilitate adherence to their rehabilitation programme. Adherence to rehabilitation programmes can be as low as 50% compromising the health advantages that can be obtained from them.^14^ In patients with osteoarthritis (OA), one of the main issues with rehabilitation and one of the reasons of small to moderate effect size into its effectiveness, has been poor adherence to prescribed rehabilitation programmes due to organisational issues including time and location, as well as patients’ everyday commitments.^15^ In addition, psychosocial variables including poor patient motivation, lack of understanding of rehabilitation exercises, self-efficacy and exercise beliefs have been related to non-adherence.^16^ ^17^ These psychosocial variables can be influenced with behavioural changes. Behavioural change theory suggests that interventions that can provide an assessment of what you want to change along with measures of your accomplishments can influence exercise adherence.^18^ Wearable technologies, by offering the possibility to monitor progress and set goals while allowing people to exercise at a time and location convenient to them could tackle the barriers to adherence particularly in relation to OA rehabilitation. Moreover this highlights one area of application, which patients were in favour of:^19^ using wearable technology as a platform to support home rehabilitation and provide progress monitor and guidance during exercising with remote feedback and support from clinicians.

A number of studies have explored the use of wearable technology in OA focusing on the clinical adoption and user preferences for portable sensors, in terms of requirements and practical implementation issues that would increase patient’s acceptance and willingness to use.^19–21^ However, there has been limited coverage of how this could be linked to psychosocial aspects of patient care. The present study is part of a larger project on patients living with OA and their preferences for a knee-monitoring device.^19^ Moving beyond design requirement and mode of use, this paper's aim is to describe psychosocial aspects as identified by patients with OA in relation to the use of wearable technologies to aid rehabilitation. The current paper is the first, to the best of our knowledge, offering another angle on patient with OA’s perspectives regarding the benefits of wearable technology on empowerment and self-management, as well as the possible impact on clinician–patient communication and shared decision-making.

## Methods

To pursue the aim of the project a qualitative study design based on focus groups was adopted. Twenty-one adults (19 females, 2 males, age range 45–65 years) suffering from OA participated to one of four focus groups after giving written informed consent. Participants were recruited from the Imperial College London NHS Trust physiotherapy departments and local communities via poster advertisements. Criteria for participation were being diagnosed with knee OA through clinical assessment or imaging, undergoing rehabilitation and having a good understanding of written and spoken English. Potential participants were excluded from the study if they presented with neurological conditions that may have influenced their cognitive function. Participants had no prior relationship with the authors before the start of the study.

The average time for the focus groups was between 45 and 60 min. Focus groups were conducted in a room of the Charing Cross Campus of Imperial College London. Two moderators (AB and EP), after introducing themselves and their roles, facilitated the discussion by following a semistructured topic guide which allowed patients views on wearable technologies to be explored.^19^ Questions like: Do you think wearable technology would help your current situation? If so, how?, Do you think it would change how you interact with clinicians? How will it affect your daily life? were asked. At the beginning of each focus group there was an introduction to the study in general, followed by a description of the focus group in terms of format and timing; this was concluded by assuring the confidentiality of personal data and allowing for question asking or possible clarifications the participants needed. At this point, before starting the debate, participants were shown the knee-monitoring device developed, although still at a prototype stage.^22^ This consisted of a sensor unit, a thin (0.2 mm) rectangular (50×10 mm) strip of flexible conductive material with two connectors at each end. The connectors were soldered via flexible cables to a small box (35×50×40 mm) which contains the circuits to allow sensor unit data capturing and Bluetooth data transmission to a proprietary device application. The sensor unit, was explained during the focus groups, could be embedded in to a pair of leggings and the box positioned in the back pocket, commonly presents in commercially available leggings.

Each focus group was audio recorded and verbatim transcribed to allow for thematic analysis at respondent level for each of the four focus groups to be conducted. This was carried out using Framework Methodology^23^ to allow development of key themes and concepts in each focus group. Patients’ responses analysis was conducted separately by the two moderators for cross-validation of the outputs from each focus group before grouping the results. Responses were compared among groups. Information saturation was reached while analysing the fourth focus group hence recruitment was ceased. Classification of patients’ responses was performed in Microsoft Excel spreadsheets.

## Results

When initially asked about their knowledge of wearable technology in OA management, it became evident that only a minority of the patients (9/21) had some awareness or could refer to a portable device. After explaining to them what wearable technologies are and providing them with some examples and by showing off the prototype, patients were invited to share their views on their use and adoption in their everyday life. Analysis of their responses suggested five overarching themes: *practical issues, utility/functionality, social impact, clinician–doctor communication and empowerment*. The first two themes have already been discussed in detail in a paper looking into design requirements of our device.^19^ The present paper presents findings on how patients felt the use of wearable technology in OA could enhance their communication with their clinicians as well as contribute to empowering them into self-managing their condition. Each theme and its subthemes are presented with relevant quotes, identified through the acronym FG and a number between 1 and 4 to indicate the focus group they came from, as well as M or F to indicate the gender of the participants, Male or Female respectively (eg, FG1, M1, etc).

### Improving communication with healthcare providers

**Figure 1:**
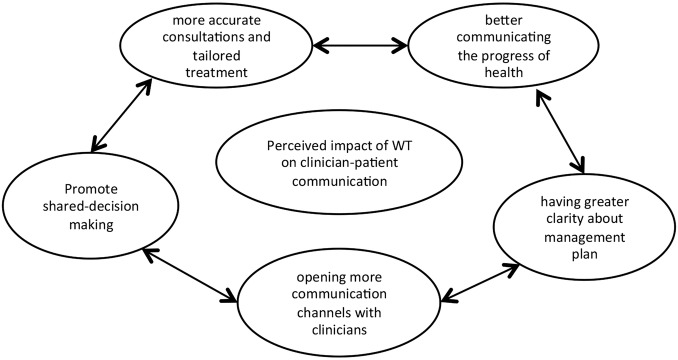
Perceived impact of wearable technology (WT) on clinician–patient communication.

It became evident that across patients there was the feeling that the use of wearable technology for the management of OA had the potential to impact on their communication with their healthcare providers, including general practitioners, surgeons and physiotherapists. This was suggested in many levels, from having more accurate consultations and tailored treatment to better communicating the progress of their health, having greater clarity about their management plan and opening more communication channels with their healthcare provider leading to shared decision-making (figure 1).

Patients felt that being able to monitor their condition could offer accurate data to their healthcare provider in an objective way, which in turn could enable them to receive not just personalised treatment but also one targeting their very specific needs. For instance, having objective evidence of the impact their condition had into their everyday life beyond their own description of it, seemed to be pivotal:

Your pain… it's very individual and very vague… such a subjective thing, because how can they assess how bad the pain is, if you're describing it to a physio or a GP, whereas if there's a piece of data which you can present which says, this is, actually this is going on in my knee, it's irrefutable, isn't it? It then can be assessed by somebody and help you know how you're doing really (FG4, F3).

Similarly and following on the benefit of having real-time individual data available, patients commented on how remote monitoring could inform clinicians on their history thus bringing into the treatment planning another level of knowledge based on their progress over time to adjust treatment accordingly; this was an aspect of treatment which was quite valuable for them, as demonstrated from the quote below:

It's not a subjective thing where you're sitting there saying, gosh, I've got a really sore knee. They can see from the data how bad it is and also how to proceed so that it's a much cleaner consultation (FG3, F4).

What was also discussed were the perceived advantages and expected improvements to their care by providing healthcare professionals additional information on their OA:

Well, it sounds like it could be a really useful addition, because it should be, as you describe it, individualised and it should be able to give the medical person hopefully a better understanding of what's going on in our body, so it does sound like it's an addition and a good thing (FG1, F5).

Another interesting finding was patients’ belief that enriching their consultations with data from the monitoring device along with their own testimony and description of symptoms would enhance the provision of accurate feedback and management planning; this also appeared to wave any elements of doubt as well as to increase their confidence in the results of the consultation:

I think it removes from me an element of doubt, have they made the right decision, is that really what I said, have they understood it properly, I think, yes, yeah… And also the reassurance that they had the real information that they needed, which, if it's just you giving it, as a layperson you put across what you think is important and you leave the doctor's surgery and you realise you didn't say X or Y or Z (FG2, F1).

Patients further discussed their view that using the knee-monitoring device had the potential to improve their interactions with their clinicians by opening more communication channels, through information sharing:

I think it would be very helpful because you would have some, you would have much the same knowledge as they had, so you could have a much clearer conversation with them (FG2, F1).

This in turn seemed to result in communicating better with their clinicians in terms of clarity of the information exchanged but also regarding the quality of advice they would receive. A more patient-centred character of consultations was evident:

I think it would make a better relationship with the clinician… because you would understand then that they're there to monitor, first of all, and advise you. They could just say to you, well that was really good there, well you could improve on that, or you could slow down on that one. So they will know the information and they'll pass on that knowledge to you where they see… (FG3, M1).

…just about every single word that a surgeon will say is just, is so important to us, and you're only, you're not seeing him too, too often, and so the, with something like that you just feel that there's more feedback, there's more information for them to see (FG2, F2).

Using the data from the monitoring device as part of their consultations was welcomed by the patients as a positive step in their care:

If it could help you communicate with health professionals like GPs and consultants that's got to be a good step forward I would think… because it would help inform you so you can actually try to work out what's happening with your knee… (FG4, F3).

Which in turn made them feel more informed, promoted shared decision-making and moved towards building a partnership with their care provider, rather than following a purely paternalistic model:

I think knowing that (data from sensor) and talking to a medical professional, you just, you feel a bit more involved in the conversation (FG2, F3).

### Patient empowerment and self-management

**Figure 2:**
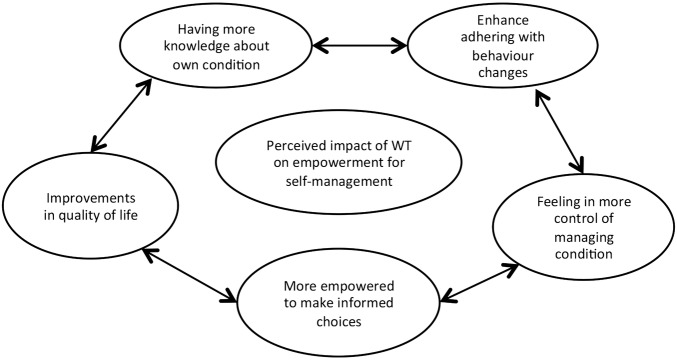
Perceived impact of wearable technology (WT) on empowerment for self-management.

Patients also expressed the view that the use of monitoring devices would benefit them, as having access to their day-to-day progress would make them feel more empowered to take control over their condition and manage it more effectively. In addition, having more knowledge about their condition as well as reinforcing what they already knew was further believed to help them adhere to a possible need for behavioural change; equally, being in control of and managing their situation in a more effective way was another manifestation of the benefits they perceived technology could offer. Finally, having the power to make informed choices about their health was associated with improvements in their quality of life, as they felt they would now also be in control, rather than just following professional guidance (figure 2).

I think the word empowerment is right because what you're looking at now is the biomechanics of walking and if I'm not doing it right and the information you give me from this study… it's going to help, it's going to benefit me. So that, I think, would be… empowerment, personal empowerment is what I strive for, that would be appropriate (FG3, M1).

Acquiring more knowledge from the data wearable technology would gather, seemed to place patients in a better position in order to introduce changes in their daily routines and stick to them; a suggested reason for that was that it was not about just providing them with information, but rather with a platform for continuous feedback on their progress:

…the information I get from the people that would tell me, and that would help me then if I need to change (FG3, M1).

So it's kind of reinforcing what you learn and helping you to remember how to do it. And then also monitoring you so it tells you if you're not doing it correctly (FG3, F1).

Having this extra bit of knowledge appeared to enable patients in acquiring a more active role in managing their health and was linked to improved outcomes, as opposed to having less information.

My feeling personally is the more I know about something that's ailing me, the more I feel I can help in some way because I'm trying to help myself get better, you know, and it's just that simple. If it's going to help me to help myself, that's where I'm at (FG3, F1).

It was further suggested that having more information and being encouraged to use a monitoring device shifted patients’ attitude from a rather reactive approach to their health condition to becoming more proactive. This appeared to also place them in a better place to communicate and negotiate their care with their healthcare provider.

Well you're going to have much more information yourself, so you can go in with this and you can be more proactive and assertive (FG4, F1).

Another advantage of using wearable technology was the potential to enhance patients’ quality of life through helping them making their preferred choices.

I think empowerment is a good word, yes, because…If you, that's me personally, I shouldn't speak for anybody else, but it seems to me that you want to do the best you can in order to live as full a life as you want to live… as you can, that you personally want to live (FG3, F1).

It's your quality of life and you're not doing your life when you're, when you've got a pain and you're laid up you're not doing the things you'd normally do, so yeah. (FG4, F1).

Another area discussed was patients’ view that having more information could increase their level of understanding, thus helping them making more informed choices:

So I think this might be able to give us more information, which I would understand rather than what we see on an X-ray for example (FG4, M1).

And this information had the potential to reinforce their ability to take control over their situation; in addition patients suggested that it would help them engage more in behavioural change or sticking to a new health regime and thus help them reach the desired heath outcome:

The thing is if we know we're doing something right, we're going to progress so much better, aren't we? (FG2, F3).

I want to live my life, so anything which can inform me, in a way it's empowering you to have more information about your health so that you can keep healthy and anything that really promotes that I'm happy to buy into quite frankly (FG4, F1).

So, if you put that patient in control of what they're doing, then it's obviously beneficial. (FG1, F3).

## Discussion

The significance of addressing the needs and views of people with long-term conditions, such as OA, in managing their health is irrefutable. This becomes even more important in light of the longer life expectancy, which ultimately means that more people will be suffering from OA and will need long-term care and management. The use of wearable technology to support people with OA could reduce the burden of this condition in each individual. Using wearable technologies for supporting chronic conditions has already been associated with improvements in patients’ quality of life.^24^ Yet, when it comes to the impact wearable technology may have on the psychosocial aspects of patients with OA's daily routine, little is known and our study aimed to address that. Our findings suggest overall positive and welcoming views in terms of promoting self-management and enhancing the clinician–patient relationship with the adoption of wearable technology for OA management. Among the areas the participants have commented on is the access to accurate real-time individualised data, having personalised feedback based on the latter, being more informed on their own health and participate in decision-making as well as communicating better with their clinicians. Other areas patients have commented on, are feeling more empowered to take control of their condition; becoming more proactive, rather than simply reactive to the doctors’ recommendations; and having increased knowledge on their OA, which could help them in making informed choices, as well as introducing and sustaining behavioural change.

The shift from passive patients, mere recipients of doctors’ advice to patients who are more proactive, informed and thus involved in the management of their own health, suggests a movement towards a more patient-centered approach from the more paternalistic models of care used in the past. Especially in long-term conditions, having sufficient knowledge not only on the condition itself and its treatment, but also on how one performs in managing it, has been paramount to self-management;^25^ and our participants’ responses supported this view.

Barlow *et al*^2^ commented on the growing body of evidence on the positive outcomes self-management of chronic conditions can have on patients, in terms of equipping them with more knowledge, helping in sustaining behavioural change, increasing their self-efficacy and improving general well-being. According to the authors, this impact becomes even more evident when compared to standard care that is, managing patients’ health without an intervention-in our case the intervention would be using the monitoring device. It could be thus proposed that the use of wearable technology seen in the above respect can indeed be beneficial in empowering patients to manage their condition and symptoms, a proposition, which is also supported by our participants’ responses.

Our participants further discussed their view that using wearable technology could open more communication channels with their healthcare professionals and promote shared decision-making. Indeed and in line with our findings, Wicks *et al*^26^ have suggested that wearable devices could succeed in promoting patient-driven care as they can encourage greater collaboration between patients and physicians, while at the same time enhancing patient involvement by understanding and using their personal data to improve their health. However, for this to happen it is crucial not just to offer patients their collected data, but also to use those as a platform to provide them with an insight into the progress of their own health and what specifically they could do to improve it. In this way and within the OA rehabilitation context, wearable devices could be used to set rehabilitation targets based on individual needs and aspirations to maintain patients’ motivation to exercise and fulfil the need of an adequate rehabilitation uptake, which is currently lacking.

In line with this, our findings suggest that the use of wearable technology can have a positive impact on adherence to rehabilitation and promote self-management. This agrees with previous studies on other chronic conditions including diabetes, hypertension, asthma and heart failure, which have suggested the same positive impact.^10^ ^11^ ^27^ ^28^ In addition, there seemed to be a willingness among our participants to use wearable technology as a tool towards assuming more control in managing their condition, supporting similar findings by Ovaisi *et al*^29^ It could be suggested that gaining control over their health could in turn lead patients to greater compliance with their rehabilitation regimes, a finding similar to what Rickerby and Woodward^30^ have found in patients with raised blood pressure. However, further research will be needed to identify patient preferences in terms of the amount of knowledge and involvement they prefer from a wearable device-supported rehabilitation approach. It is worth mentioning that this would also require relative acceptance and engagement from the healthcare professionals,^31^ since it will be the latter who will carry the weight of not just prescribing treatment or rehabilitation programmes, but having an active role in educating their patients.

We recognise that all our participants came from the London area, and although we are confident about the representativeness of findings within our sample, further research including other geographical areas could offer an insight to views of patients who are possibly less exposed to wearable technologies. Moreover, we acknowledge that gender representation is not uniform in this study, 19 females against 2 males, and this may limit generalisation of the findings but this reflects the fact that OA has a higher prevalence in women. Another limitation of our study is that at the time of the project, the participants had not tried yet the wearable technology; therefore their views are based on their expectations of what the technology could offer and experimental data will be needed to confirm these views. It would be worth following participants up and explore their views at a later stage after they had the opportunity to use the knee monitoring device alongside the investigation of its clinical use to verify its potential in enhancing OA management.

In spite of these limitations, this study should encourage the development of technology as a rehabilitation tool which agrees to patients’ preferences and foster new studies to explore the effects of its adoption in clinical domains to be able in the future to improve patient with OA's outcomes and experience.

Overall our findings on patients’ views on wearable technology for the management of OA have been positive, encouraging and worth taking into account; nevertheless for this positive attitude to continue, it is vital that the emphasis continues to be placed on user preferences and the way patients perceive new technologies will help them towards a more holistic approach including psychosocial consequences and lifestyle changes.^2^
